# Knockdown of TOP2A reverses cisplatin resistance in ovarian cancer by inhibiting EMT via ferroptosis mediated by the TP53/GPX4/SLC7A11 axis

**DOI:** 10.3389/fimmu.2025.1675373

**Published:** 2025-10-29

**Authors:** Xinyue Liu, Junxia Wang, Yangyu Liu, Yan Han, Zhichao Qin, Yu Wang, Shanxiang Gao, Xinghua Li, Yuping Suo

**Affiliations:** ^1^ The Gynecology Department of Shanxi Provincial People Hospital, Shanxi Medical University, Taiyuan, China; ^2^ Institute of Upper Gastrointestinal Tumour Prevention and Treatment, Shanxi Medical University, Changzhi, China; ^3^ The Pathology Department of Shanxi Provincial People Hospital, Shanxi Medical University, Taiyuan, China

**Keywords:** TOP2A, cisplatin resistance, ovarian cancer, ferroptosis, EMT, TP53

## Abstract

**Introduction:**

Cisplatin resistance is a major challenge in ovarian cancer therapy, particularly for high-grade serous ovarian cancer (HGSOC). DNA topoisomerase IIα (TOP2A) relates to cancer drug resistance, yet its role and molecular mechanisms in ovarian cancer cisplatin resistance remain unclear.

**Methods:**

TOP2A expression was detected in HGSOC tissues and cisplatin-resistant cells (SKOV3-DDP, OVCAR3-DDP). Functional assays combined with TOP2A knockdown evaluated cisplatin sensitivity and malignant phenotypes (proliferation, invasion, migration) in resistant cells. RNA-seq and GEO cisplatin resistance dataset (GSE214302) validated TOP2A’s role in cisplatin resistance, prognostic value, and associations with TP53, GPX4, SLC7A11. Molecular docking/Co-IP confirmed TOP2A-TP53 interaction. Fer-1 and TP53 knockdown clarified TP53/GPX4/SLC7A11 axis regulation of ferroptosis and EMT, and an *in vivo* xenograft tumor model validated these findings.

**Results:**

TOP2A is highly expressed in HGSOC tissues and cisplatin-resistant cells, with high levels strongly associated with tumor progression (advanced stage, high grade, lymph node metastasis) and poor prognosis. RNA-seq shows TOP2A correlates with TP53, GPX4, SLC7A11.GEO dataset analysis confirms all four associate with cisplatin resistance. SLC7A11 and TOP2A are effective resistance biomarkers, and high TOP2A predicts shorter progression-free survival. Molecular assays verify direct TOP2A-TP53 interaction. Functional experiments reveal TOP2A knockdown enhances cisplatin sensitivity, inhibits malignancy, activates ferroptosis, and suppresses EMT via the TP53/GPX4/SLC7A11 axis. This effect is reversed by Fer-1 or TP53 knockdown, with mechanisms validated *in vivo*.

**Conclusion:**

TOP2A represents a potential prognostic biomarker and therapeutic target for cisplatin resistance in ovarian cancer (OC), as it regulates ferroptosis and EMT via the TP53/GPX4/SLC7A11 axis, which is mediated by its direct interaction with TP53. This thus provides a novel direction for treating cisplatin-resistant OC.

## Introduction

Ovarian cancer(OC) is the most lethal gynecological malignancy, with approximately 70% of patients being diagnosed at advanced stages (1). According to epidemiological predictions for 2025, the global burden of OC remains substantial, with approximately 20,890 new cases and 12,730 deaths projected in the United States alone (2). Cisplatin, a platinum-based chemotherapeutic agent approved by the U.S.Food and Drug Administration(FDA),is widely used in the treatment of OC (3). However, 65% to 80% of patients develop disease recurrence, which is accompanied by either primary or acquired cisplatin resistance (4). Platinum resistance has emerged as a major barrier to the effective treatment of OC (5). For patients with cisplatin-resistant ovarian cancer, the objective response rate (ORR) to platinum-free single-agent chemotherapy, such as paclitaxel and etoposide, is only 20–28%, accompanied by significant toxicity and side effects (6).Therefore, identifying novel drug resistance drivers and elucidating their targetable mechanisms represent the key to overcoming the bottleneck in ovarian cancer treatment.

TOP2A, a DNA topoisomerase encoded by a gene on chromosome 17q21.2 with 36 exons, induces DNA double-strand breaks and rejoining, playing a role in DNA transcription, replication, chromatin condensation, and chromatid separation (7). Over the past three decades, TOP2A has garnered substantial research attention due to its functional role in human malignancies. TOP2A is highly expressed in proliferating cells, and its upregulation is closely associated with a broad range of cancers (8), including OC (6, 9). Clinically used TOP2A inhibitors commonly include etoposide and doxorubicin (10). Studies have demonstrated that TOP2A mRNA expression is significantly elevated in platinum-resistant patients. In recurrent OC patients who have previously received platinum-based combination chemotherapy, weekly cisplatin administration combined with daily etoposide treatment is threefold more effective than conventional therapeutic regimens (11). TOP2A holds promise as a potential biomarker for predicting resistance to pegylated liposomal doxorubicin (PLD) and for prognosticating patient clinical outcomes (12–14). However, current studies on the specific functional role and molecular mechanisms of TOP2A in cisplatin resistance of ovarian cancer remain in the preliminary exploration stage. Whether its regulatory effects involve core pathways associated with drug resistance has not yet been clarified.

With the in-depth investigation of drug resistance mechanisms, ferroptosis and epithelial-mesenchymal transition (EMT) have been identified as key biological processes underlying platinum resistance in ovarian cancer (15–18). Among these, ferroptosis is driven by intracellular iron accumulation and lipid peroxidation, and is precisely regulated by antioxidant enzymes such as glutathione peroxidase 4 (GPX4) (19–23). EMT, on the other hand, is a core program that enables tumor cells to acquire invasive and metastatic capabilities. It drives the transition of cells from an epithelial to a mesenchymal phenotype via signaling axes such as transforming growth factor-β (TGF-β), hypoxia-inducible factor-1α (HIF-1α), and Notch, thereby directly impairing the cytotoxic effect of cisplatin on tumor cells (17, 18). Current functional studies have mostly focused on downstream nodes. For instance, the SLC7A11 inhibitor sulfasalazine restores tumor cell sensitivity to platinum-based drugs by increasing lipid peroxidation levels in colorectal cancer (24). Similarly, TPI1 gene silencing exerts the same effect in oral squamous cell carcinoma (25), and downregulation of MPC1 expression achieves this outcome in head and neck cancer (26, 27). Notably, cross-cancer studies have provided clues regarding the association between TOP2A and drug resistance pathways. In cutaneous squamous cell carcinoma, TOP2A expression is positively correlated with ferroptosis sensitivity (28).In hepatocellular carcinoma, the small molecule Phillyrin can specifically inhibit TOP2A, block the JAK2/STAT3 signaling axis, and downregulate GPX4 expression, thereby enhancing ferroptosis (29).Additionally, bioinformatics analyses have identified TOP2A as a ferroptosis-resistance regulatory hub (30).However, these studies only focus on the single pathway of ferroptosis and do not involve EMT. Furthermore, none of them have been validated in ovarian cancer, which makes it impossible to clarify the role of TOP2A in cisplatin resistance of ovarian cancer (31, 32), suggesting functional crosstalk between the two pathways.However, in OC, direct evidence is still lacking regarding whether targeting a single key factor can simultaneously activate ferroptosis and block EMT, thereby reversing cisplatin resistance.More importantly, whether TOP2A is involved in the aforementioned crosstalk constitutes a critical gap in the current research field.

Based on the aforementioned research status, this study conducted a systematic investigation focusing on “TOP2A-mediated regulation of cisplatin resistance in OC”. First, it verified that TOP2A is highly expressed in ovarian cancer tissues and cisplatin-resistant OC cell lines, and this high expression is associated with poor prognosis in patients.Second, via RNA sequencing(RNA-seq) and mechanistic validation, this study is the first to identify the “TOP2A/TP53/GPX4/SLC7A11” regulatory axis, defining its role in coupling ferroptosis induction with (EMT inhibition. Third, analysis of public ovarian cancer genomic data from the Gene Expression Omnibus (GEO) database demonstrated that TOP2A, TP53, GPX4, and SLC7A11 exhibit differential expression between cisplatin-resistant and cisplatin-sensitive OC cohorts. This analysis further confirmed that high TOP2A expression is associated with shorter patient progression-free survival, while receiver operating characteristic (ROC) curve analysis showed that TOP2A possesses moderate diagnostic efficacy. Finally, functional experiments demonstrated that TOP2A binds directly to TP53.TOP2A knockdown restores cisplatin sensitivity by activating TP53-dependent ferroptosis and reversing EMT.

## Materials and methods

### Tissue specimens

The tissue samples in this study were obtained from 75 patients with primary HGSOCadmitted to Shanxi Provincial People’s Hospital Affiliated to Shanxi Medical University between 2021 and 2024, as well as 15 patients who underwent bilateral salpingo-oophorectomy due to non-ovarian diseases (adenomyosis and uterine fibroids).Among these HGSOC patients, the mean age was 55.32 ± 9.6 years (range: 35–68 years). According to the International Federation of Gynecology and Obstetrics (FIGO) staging system, 22 patients were at stage I–II and 53 at stage III–IV. For histological grading, 25 cases were grade G1, and 50 cases were grades G2–G3 (samples of grades G2 and G3 were combined for statistical analysis).All ovarian cancer tissues were collected at the time of surgical resection.The inclusion criteria for HGSOC patients were as follows: (1) Confirmed diagnosis of HGSOC by pathological examination of surgical specimens; (2) Postoperative receipt of standardized platinum-based combination chemotherapy (cisplatin plus paclitaxel regimen, 4–6 cycles in total) in accordance with clinical guidelines.The inclusion criteria for HGSOC patients were as follows: (1) Confirmed diagnosis of HGSOC by pathological examination of surgical specimens; (2) Postoperative receipt of standardized platinum-based combination chemotherapy (cisplatin plus paclitaxel regimen, 4–6 cycles in total) in accordance with clinical guidelines.All tissues were processed into sections for immunohistochemical (IHC) staining, which was performed to evaluate the expression level of TOP2A. Staining results were then assessed by two experienced pathologists using a standardized scoring system.This study collected complete pathological and clinical data of all patients, and all participants signed written informed consent forms prior to the study.The study protocol strictly adhered to the Declaration of *Helsinki* and was approved by the Ethics Committee of Shanxi Provincial People’s Hospital Affiliated to Shanxi Medical University (Approval No.: 776).

### Immunohistochemistry

Immunohistochemical (IHC) staining was performed using the streptavidin-peroxidase method to detect the expression of TOP2A and TP53 in ovarian tissue and xenografts. Paraffin-embedded tissue sections (4 μm) were deparaffinized, rehydrated, and subjected to antigen retrieval with citrate buffer. Sections were incubated with a primary antibody against TOP2A (1:200, T55008,Abmart) or TP53(1:200,TA0879,Abmart)overnight at 4 °C, followed by incubation with a secondary antibody (PV9001, ABGH-Bio) for 15 min at room temperature. Sections were then stained with DAB (C-0003, Bioss) and mounted. A semi-quantitative scoring system was used to evaluate the sections: intensity (0–3 points) × area percentage (0-0.9 points). A total score of ≥0.6 was considered high expression, and ≤0.45 was considered low expression.

### Cell culture and cell transfection

Epithelial OC cell lines SKOV3, A2780, and OVCAR3, as well as their cisplatin-resistant counterparts SKOV3/DDP, A2780/DDP, and OVCAR3/DDP, were purchased from Shanghai Jinyuan Co., Ltd., a company specializing in human cell products. The human ovarian surface epithelial cell line OSE was provided by the Laboratory of Experimental Gynecologic Oncology, Chinese Academy of Medical Sciences. Prior to use, all human cell lines underwent standard mycoplasma contamination screenings and were authenticated using STR profiles. Culture conditions for the cell lines included 37 °C, 5% CO2, and DMEM medium with 10% fetal bovine serum (FBS) from Cell-box in China. To maintain drug resistance, the culture medium of resistant strains was supplemented with 0.5 μg/ml cisplatin (HY-17394, MCE). The incubator was disinfected daily with ultraviolet light for at least 30 minutes. The culture medium was changed regularly, and cells were passaged when confluence reached 80%. Traditional methods were employed to stably transduce cells with packaged NC vectors and lentiviral GV493 vectors carrying shRNA-TOP2A and shRNA-TP53. siRNA and The RNA lentivirus was obtained from Shanghai Bioson Biotechnology Co., Ltd. and Haixing Biotechnology Co., Ltd. (Sequences are listed in **Supplementary Table S1**). One week prior to transfection, the culture medium was replaced with one devoid of cisplatin. For transient transfection, 5 µL of Lipofectamine™ 3000 (L3000015, Thermo Fisher Scientific) was used per well in a 6-well plate. NC and siRNA(shRNA) groups were established by seeding SKOV3/DDP and OVCAR3/DDP cells in 6-well plates and culturing them at 37 °C for 24 hours. The working concentration of siRNA was 50 nM.The volumes of NC and shRNA lentivirus were calculated based on cell numbers and added to the cells (SKOV3/DDP: MOI = 10; OVCAR3/DDP: MOI = 10). Subsequently, the cells were incubated at 37 °C for 24 hours, after which the medium was replaced with fresh complete medium. Fluorescence microscopy was used to assess successful cell transduction 48 to 72 hours post-transduction, indicated by green fluorescence. Stable transductants were selected using 2μg/ml puromycin(ST551, Beyotime). Subsequently, knockdown effectiveness was determined by Real-Time PCR and western blot (WB) analysis.

### Cell counting kit-8 assay

The CCK-8 assay was used to measure cell viability. Cells in logarithmic growth phase were collected and seeded at a density of 8×10³ cells per well in a 96-well plate (100μL per well) and cultured overnight. Each group was replicated three times and treated with various concentrations of cisplatin (0, 1, 2, 4, 8, 10 μg/ml) or ferrostatin-1(fer-1; HY-100579, MCE) for 48 hours. Ten microliters of CCK8 solution (100-106, GOONIEBIO) was added to each well at pre-arranged intervals, and two hours after that, the absorbance at 450 nm was measured with a multi-scan spectrophotometer.

### Colony formation assay

A 6-well plate containing 700 transfected cells was cultured for around 10 days in a medium containing 10% FBS for this test. Staining the colonies with 0.1% crystal violet for an additional 30 minutes followed 30 minutes of fixing in 4% paraformaldehyde. We used ImageJ to examine high-resolution photos of the colonies.

### Transwell assays

To measure the OC cells’ ability to invade, these tests made use of transwell membranes that were covered with Matrigel (Corning 3422, 8 μm pore size). At first, 200 μl of DMEM without FBS was added to the upper chamber, where 2-4 × 104 cells were inserted, and 500 μl of DMEM with 10% FBS was added to the lower chamber. The chambers were incubated at 37 °C for 24 or 48 hours, then rinsed with PBS. After that, they were fixed with 4% paraformaldehyde for approximately 30 minutes. The cells on the upper side of the membrane were then removed using a cotton swab. Following a 30-minute staining period, the cells were washed with double-distilled water and allowed to dry before imaging. The membranes were subsequently rinsed with crystal violet.

### Wound healing assay

The purpose of this experiment was to measure the OC cells’ migratory capabilities. A sterile 10 μl pipette tip was used to make a scratch when the cells in a 6-well plate had attained 100% confluence. The cells were subsequently cultured in DMEM devoid of FBS and their migration inside the wound was tracked by taking electron microscopy pictures at 0 and 48 hours. Measuring the change in wound area allowed us to quantify the degree of cell migration.

### Real-time PCR

Cells were cultured in 6-well plates. Total RNA extraction and concentration measurement were performed for each group as per the kit’s protocol (AG21023, SteadyPure). Reverse transcription was conducted using an oligo(dT) primer at 42 °C for 60 minutes, followed by 70 °C for 5 minutes. For qPCR with SYBR Green dye, the thermal cycling conditions included an initial denaturation at 95 °C for 30 seconds, then 40 cycles of 95 °C for 15 seconds and 60 °C for 30 seconds. A melting curve analysis was used to verify the specificity of the PCR products. The cycle threshold (Ct) values of the genes were recorded, and relative mRNA expression levels were determined using the 2−ΔΔCt method. First-strand cDNA synthesis kit (No. g592) and BlasTaq 2X qPCR MasterMix (No. g891) were purchased from Applied Biological Materials. The qPCR primers used are listed in **Supplementary Table S2**.

### Western blot

Cells were lysed using RIPA buffer (P0013B, Beyotime) supplemented with phenylmethylsulfonyl fluoride (PMSF) and kinase inhibitors (P1005, Beyotime) to extract proteins. Protein concentrations were measured using a BCA protein assay kit (ZJ102, EPIZYME BIOTECH. 20 ug Proteins were separated using 10% SDS-PAGE(SW243-02,SEVEN BIOTECH) and then transferred onto 0.45μm PVDF membranes(Millipore). Following a 2-hour blocking step with 5% skim milk, the membranes were incubated with primary antibodies at 4 °C overnight and HRP-conjugated secondary antibodies for 1 hour. The binding of antibodies was visualized using ECL (enhanced chemiluminescence) reagents (G2074 and G2014, Servicebio). The primary and secondary antibodies used are detailed in **Supplementary Table S3**.

### RNA-sequencing

Total RNA was isolated from transfected cells with silenced and NC cells using TRIzol reagent (Hyclone), and sent to Biotree Technology Company (Shanghai, China) for RNA-seq analysis according to the standard Illumina protocol. Double-stranded cDNA (250–300 bp) was size-selected using AMPure XP beads, followed by PCR amplification, purification, and library construction. Library quantification was performed using Qubit, and insert size was assessed by Agilent 5400. After confirming the insert size met expectations, the effective library concentration was accurately quantified by qPCR (library concentration >2 nM) to meet high-quality library standards.Libraries with different index sequences were pooled in proportion, followed by Illumina Novaseq PE150 sequencing. Data analysis was performed in the R programming environment. Differential expression analysis was conducted using the DESeq2 package, with the threshold for significantly differentially expressed genes set at |log2FoldChange| > 1.0 and padj < 0.05. Subsequently, DAVID was used to perform Gene Ontology (GO) term and Kyoto Encyclopedia of Genes and Genomes (KEGG) pathway enrichment analyses on differentially expressed genes (DEGs).

### Bioinformatics analysis

Sequencing data from the GSE214302 microarray were downloaded from the GEO database. Gene expression profiles of two sample groups (cisplatin-sensitive and cisplatin-resistant) were examined, and differential genes were analyzed using the limma package. The volcano plots were generated with the screening criteria of | log_2_FC|>1 and adjusted P<0.05).The Heatmap package was used to generate standardized expression heatmaps for TOP2A and other drug resistance-related genes. For the genes TOP2A, TP53, GPX4, and SLC7A11, box plots combined with the Wilcoxon rank-sum test were applied to analyze intergroup differences in expression. ROC curves (with area under the curve [AUC] values) were used to evaluate the diagnostic efficacy of these four genes. Patients were stratified based on TOP2A expression levels, and differences in progression-free survival (follow-up unit: months) were analyzed using the Kaplan-Meier method (Log-rank test).

### Molecular docking analysis

Protein-protein rigid docking was performed using GRAMM software (conformations of ligand and receptor molecules remained unchanged, with only the optimal binding sites searched on the protein surface). Based on the names of target genes, the amino acid sequences of TOP2A and TP53 were retrieved from the UniProtKB database. These sequences were input into the SWISS-MODEL server for homology modeling, and the Protein Data Bank (PDB) file of the optimal protein structure was screened and downloaded. The PDB file was then imported into GRAMM software for molecular docking calculations. The top 10 docking results were collected, and the result with the highest score (ranked first) was selected as the optimal binding mode for subsequent analyses.

### Co-immunoprecipitation

Co-IP assay was performed to verify the binding properties between proteins. Cisplatin-resistant ovarian cancer cell lines (SKOV3-DDP and OVCAR3-DDP) were selected and cultured to 80–90% confluency. Cells were lysed on ice using RIPA lysis buffer (HYP103, HYCEZMBIO) supplemented with protease/phosphatase inhibitors. After shaking at 4 °C for 30 minutes, the lysate was centrifuged at 12,000 rpm for 20 minutes to collect the supernatant. A portion of the supernatant was mixed with rabbit anti-human TOP2A primary antibody, with normal rabbit IgG (B30011, Abmart) as the negative control, and incubated overnight at 4 °C. The IP complex was then incubated with Protein A/G Plus agarose beads (Santa Cruz) on a multi-functional rotary shaker at 4 °C for 5 hours, followed by centrifugation. The resulting bead-antibody-antigen complex was washed three times with lysis buffer containing a mixture of protease and phosphatase inhibitors, and the supernatant was collected after centrifugation. Finally, the complex was mixed with an equal volume of 1× loading buffer, boiled at 95 °C for 5 minutes, and analyzed by WB.

### Mitochondrial membrane potential, reactive oxygen species and lipid peroxidation assays

To evaluate the levels of MMP, ROS and LPO in cells, we used the JC-1 Mitochondrial Membrane Potential Detection Kit (G1515, Servicebio), DCFH-DA (HY-D0940, MCE), and BODIPY 581/591 C11 (G1733, Servicebio). Briefly, cells in 6-cm dishes were incubated with JC-1 fluorescent probe, 10 μM DCFH-DA, and 10 μM BODIPY 581/591 C11 for 30 minutes. Finally, fluorescence microscopy(Mateo FL, Leica)was used to analyze the levels of ROS, MMP and LPO in cells.

### Transmission electron microscopy

For TEM analysis, cell samples were initially fixed with 2.5% glutaraldehyde and subsequently post-fixed with 1% osmium tetroxide. They were then dehydrated with a graded series of acetone (30%, 50%, 70%, 80%, 90%, 95%, 100%), with the 100% concentration changed three times, for 15 minutes each. Following this, samples were infiltrated with a mixture of the dehydrating agent and Epon-812 embedding medium in ratios of 3:1, 1:1, and 1:3, respectively, before being embedded in pure Epon-812. Ultrathin sections (60–90 nm) were cut using an ultramicrotome and mounted on copper grids. Sections were stained with uranyl acetate for 10–15 minutes, followed by lead citrate for 1–2 minutes, both at room temperature, before being examined by TEM (JEM-1400FLASH, JEOL).

### Superoxide dismutase, glutathione, lactate dehydrogenase and lipid peroxidation malondialdehyde assays

To measure the levels of SOD, GSH, LDH, and MDA in cells, we used the Total SOD Assay Kit with WST-8 (S0101S, Beyotime), GSH Detection Kit (G4305, Servicebio), LDH Cytotoxicity Assay Kit with WST-8 (C0018S, Beyotime), and MDA Assay Kit (A003-1-2, Nanjing Jiancheng Bioengineering). Briefly, cells in 6-cm dishes were processed according to the manufacturer’s protocol, and absorbance was measured at 450 nm,412nm or 532 nm.

### *In vivo* studies

This study has received the necessary ethical approvals from Shanxi Medical University’s Fifth Clinical Medical College’s Ethics Committee (Approval No. 776). A subcutaneous xenograft tumor model was established in 5-week-old female BALB/c nude mice using 5000000 SKOV3/ddp cells (mice provided by Sibeifu (Suzhou) Biotechnology Co., Ltd., Production License No. SKXK (Su) 2022-0006). The mice were divided into four groups (n = 6 per group): shNC, shTOP2A, shTP53, and shTOP2A+shTP53. All xenografted mice were intraperitoneally injected with DDP (4 mg/kg) every 6 days (1). After 28 days, the mice were euthanized by cervical dislocation, and tumors were collected for analysis. Tumor volume was calculated using the formula (length × width²)/2, and tumor diameter was monitored throughout the experiment to ensure it remained below 15 mm.

### Statistical analysis

Statistical analyses were performed using GraphPad Prism 10.1.2, with data derived from at least three independent experiments. Results were presented as mean ± standard deviation (SD). Differences between groups were evaluated using Student’s t-test and chi-square (χ²) test. A two-tailed P value < 0.05 was considered statistically significant.(*p < 0.05, **p < 0.01,***p < 0.001).

## Results

### TOP2A in OC is elevated and associated with prognosis

**Figure 1:**
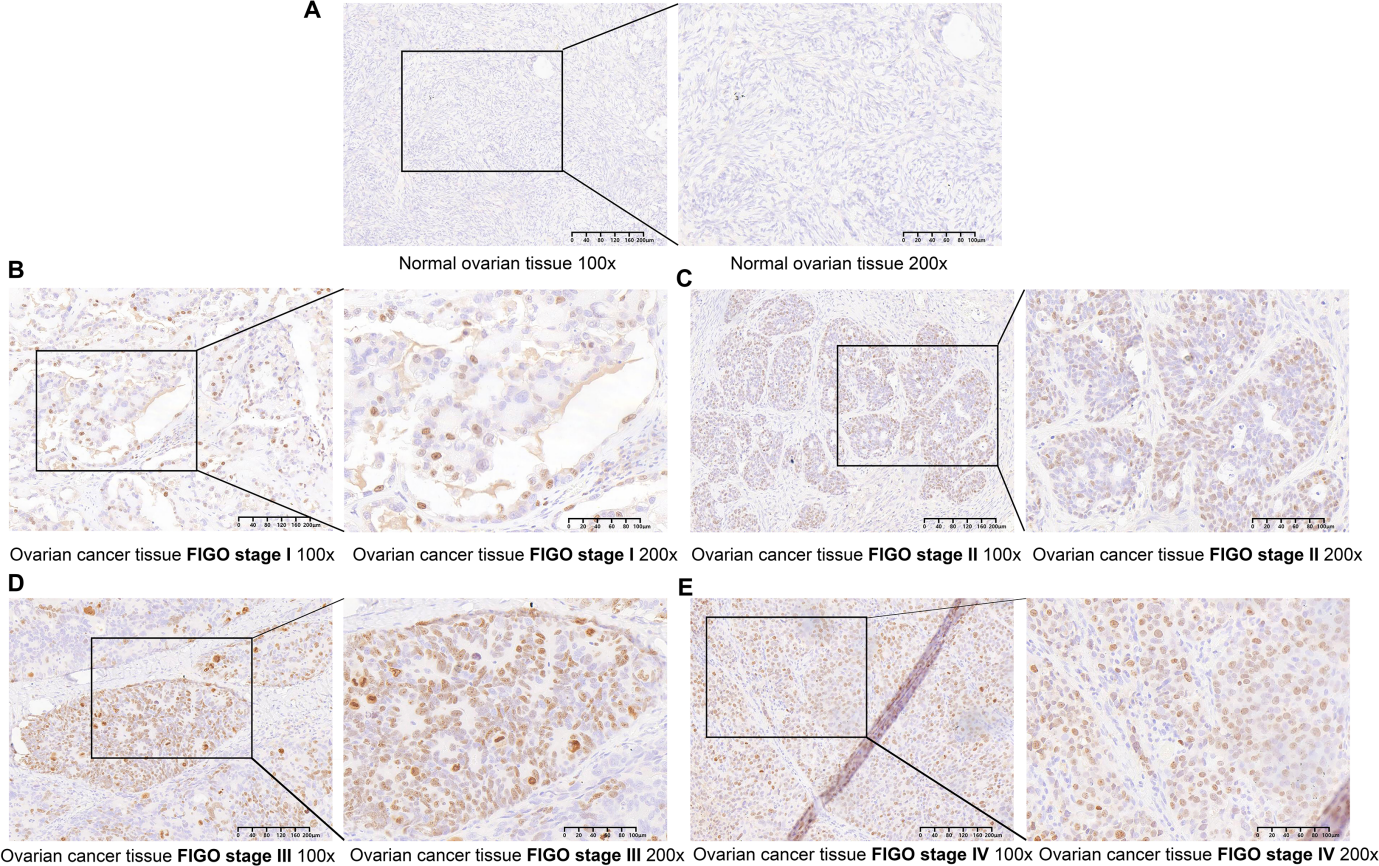
TOP2A is upregulated in OC **(A)** TOP2A was negative in normal ovarian tissues. **(B-E)** TOP2A is differentially expressed in EOC across different FIGO stages (I-IV)(×100, 200μm;×200, 100μm).

We conducted immunohistochemical staining on 75 pathological sections from EOC patients at various stages and 15 normal ovarian tissue samples, followed by clinical data analysis.Findings revealed that TOP2A was highly expressed in EOC tissues, predominantly exhibiting granular or diffuse nuclear distribution (**Figure 1**). In the sample cohort, 32 cases exhibited low TOP2A expression, while 43 cases showed high expression.TOP2A high expression rate in primary EOC tissues was 85.3% (64/75), significantly higher than that in normal ovarian tissues [20.00% (3/15), ***p < 0.001].TOP2A expression levels were positively correlated with tumor stage (*p = 0.018), histological grade (**p = 0.002), and lymph node metastasis (*p = 0.035).Increased TOP2A expression was associated with advanced tumor stage, higher histological grade, and increased lymph node metastasis incidence.However, no significant correlation was observed between TOP2A expression and patient age (*p* = 0.365) (**Table 1**).

**Table 1:** Relationship between TOP2A protein expression and clinicopathological features of Epithelial ovarian cancer.

Clinicopathological feature	n	Low(%)	High(%)	X^2^	*p* Value
Age (years)				0.82	0.365
≤50	35	13	22		
>50	40	19	21		
FIGO stage				5.63	0.018^*^
I-II	22	14	8		
III-IV	53	18	35		
Histological grade				9.74	0.002^**^
G1	25	17	8		
G2-G3	50	15	35		
Lymph node metastasis				4.44	0.035^*^
–	34	19	15		
+	41	13	28		

*p<0.05, **p<0.01.

### TOP2A is highly expressed in cisplatin-resistant OC

**Figure 2:**
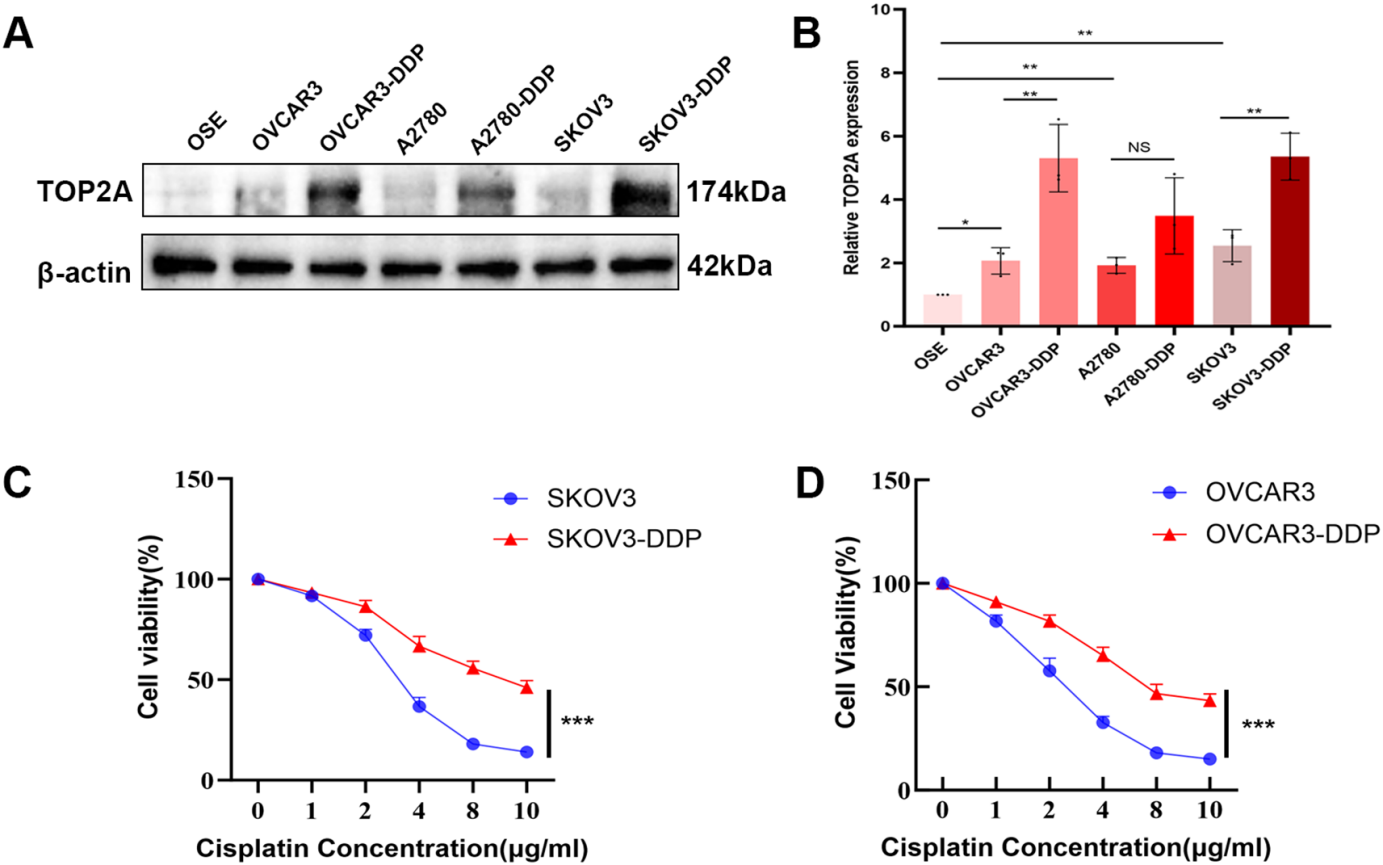
TOP2A is highly expressed in cisplatin-resistant OC cells. **(A)** WB analysis of TOP2A protein expression in EOC cells (OVCAR3, A2780, SKOV3), their cisplatin-resistant derivatives (OVCAR3-DDP, A2780-DDP, SKOV3-DDP), and OSE. **(B)** Quantitative analysis of TOP2A expression levels from **(A). (C)** Cisplatin sensitivity curves of SKOV3 and SKOV3-DDP cells, determined by CCK-8 assay following 48-hour treatment with gradient cisplatin concentrations (0, 2, 4, 6, 8, 10 μg/ml). IC50 values are indicated: SKOV3 (3.2 μg/ml) and SKOV3-DDP (8.9 μg/ml). **(D)** Cisplatin sensitivity curves of OVCAR3 and OVCAR3-DDP cells under the same treatment conditions as in **(C)**, with IC50 values: OVCAR3 (2.6 μg/ml) and OVCAR3-DDP (7.4 μg/ml). All experiments were performed with 3 independent biological replicates (n=3), and each biological replicate included 3 technical repeats. Data are shown as mean ± SD. Statistical significance: *p < 0.05, **p < 0.01, ***p < 0.001. Not Significant:NS.

To explore the association between TOP2A and the malignant behaviors as well as chemoresistance of OC, WB was used to detect TOP2A expression in EOC cells, their cisplatin-resistant derivatives (OVCAR3/OVCAR3-DDP, A2780/A2780-DDP, SKOV3/SKOV3-DDP), and normal ovarian epithelial cells (OSE).As shown in **Figures 2A, B**, TOP2A exhibited high expression in EOC cells compared with OSE cells. Furthermore, TOP2A was significantly overexpressed in SKOV3-DDP and OVCAR3-DDP cells compared with their respective cisplatin-sensitive parental cell lines. However, there was no significant difference in the protein expression level of TOP2A between A2780-DDP cells and their cisplatin-sensitive counterparts (**Figures 2A, B**). The CCK-8 assay was employed to determine the optimal cisplatin concentration for cell culture and verify the chemoresistance of ovarian cancer cells. After 48 hours exposure to gradient cisplatin concentrations (0, 2, 4, 6, 8, 10 μg/ml), the viability of SKOV3 and OVCAR3 cells was significantly lower than that of SKOV3-DDP and OVCAR3-DDP cells (SKOV3: IC50 = 3.2 μg/ml; SKOV3-DDP: IC50 = 8.9 μg/ml; OVCAR3: IC50 = 2.6 μg/ml; OVCAR3-DDP: IC50 = 7.4 μg/ml) (**Figures 2C, D**). These results confirm that SKOV3-DDP and OVCAR3-DDP cells exhibit significant cisplatin resistance. Moreover, a cisplatin concentration of 3.0 μg/ml was determined to be suitable for subsequent cell function experiments.

### TOP2A silencing enhances cisplatin sensitivity in cisplatin-resistant OC Cells

**Figure 3:**
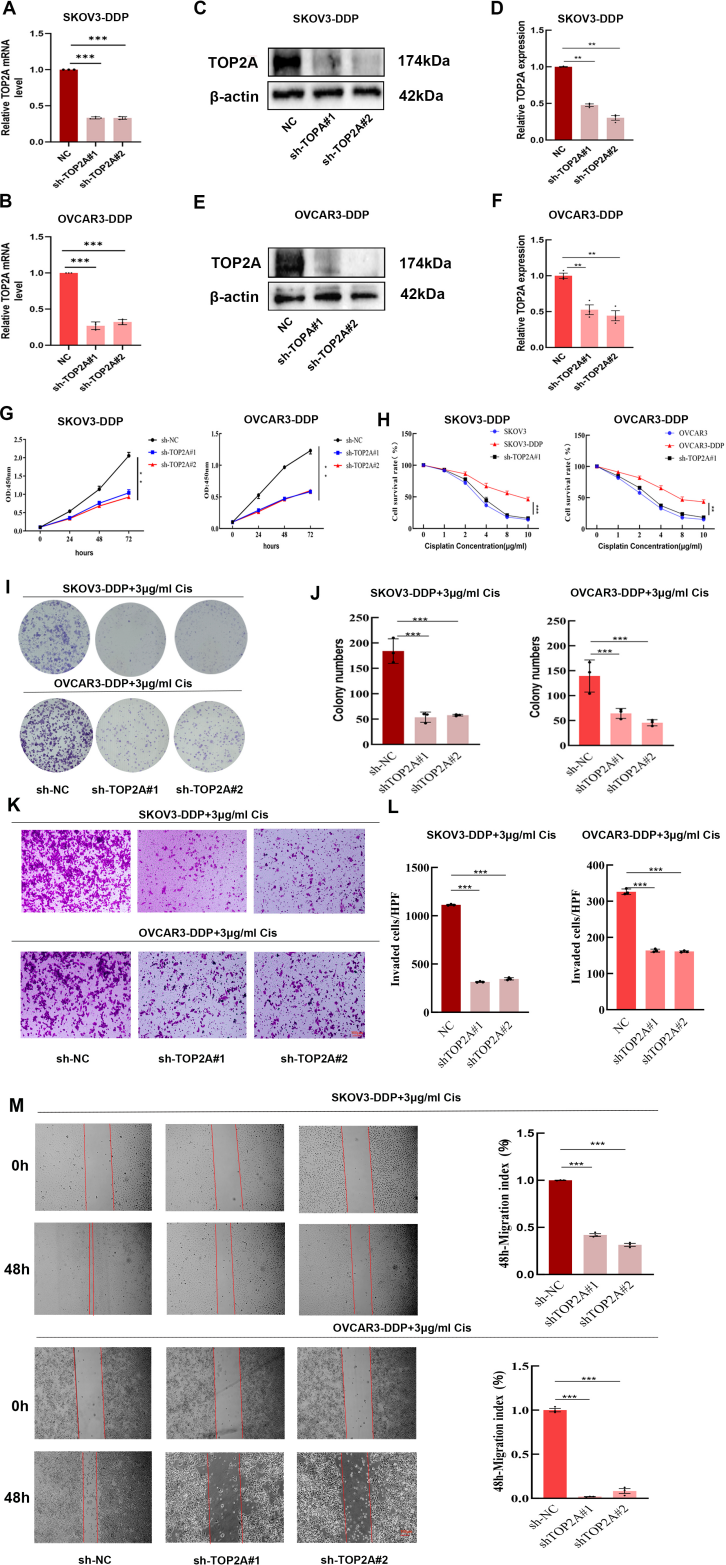
TOP2A promotes cisplatin resistance in OC cells. **(A-F)** qPCR **(A,B)** WB **(C,E)** confirm TOP2A knockdown in SKOV3-DDP and OVCAR3-DDP cells. Quantitative analysis of the WB is presented in **(D, F)**. **(G-M)** The proliferation, invasion, and migration capacities of TOP2A-knockdown SKOV3-DDP and OVCAR3-DDP cells were assessed using CCK-8 assays **(G, H)**, colony formation assays **(I, J)**, Transwell assays **(K, L)**, and wound healing assays **(M)** under 3 μg/ml cisplatin treatment. All experiments were performed with 3 independent biological replicates (n=3), and each biological replicate included 3 technical repeats. Data are shown as mean ± SD. Statistical significance: *p < 0.05, **p < 0.01, ***p < 0.001.

Stable TOP2A knockdown in cisplatin-resistant SKOV3-DDP and OVCAR3-DDP cells was achieved through shRNA transfection. qPCR and WB analyses confirmed significant reductions in TOP2A expression at both mRNA and protein levels compared to negative controls (**Figures 3A-F**). CCK-8 assays showed that TOP2A knockdown significantly inhibited the proliferation of SKOV3-DDP and OVCAR3-DDP cells compared to the NC group (**Figure 3G**). After 48-hour treatment with gradient cisplatin concentrations (0-10 μg/ml), TOP2A-knockdown SKOV3-DDP and OVCAR3-DDP cells exhibited significantly enhanced cisplatin sensitivity compared to their parental resistant counterparts (**Figure 3H**). Subsequently, TOP2A-knockdown SKOV3-DDP and OVCAR3-DDP cells were used in experiments with 3 μg/ml cisplatin treatment.Colony formation assays confirmed results consistent results with the CCK-8 assay (**Figures 3I, J**). The invasive and migratory capacities of the cells were further evaluated using Transwell and wound healing assays.Results demonstrated that TOP2A-knockdown cells exhibited significantly decreased invasion and migration rates (**Figures 3K-M**). These findings indicate that TOP2A may contribute to cisplatin resistance in OC cells *in vitro*.

### The TOP2A-TP53-ferroptosis axis synergistically regulates cisplatin resistance and prognosis in OC

**Figure 4:**
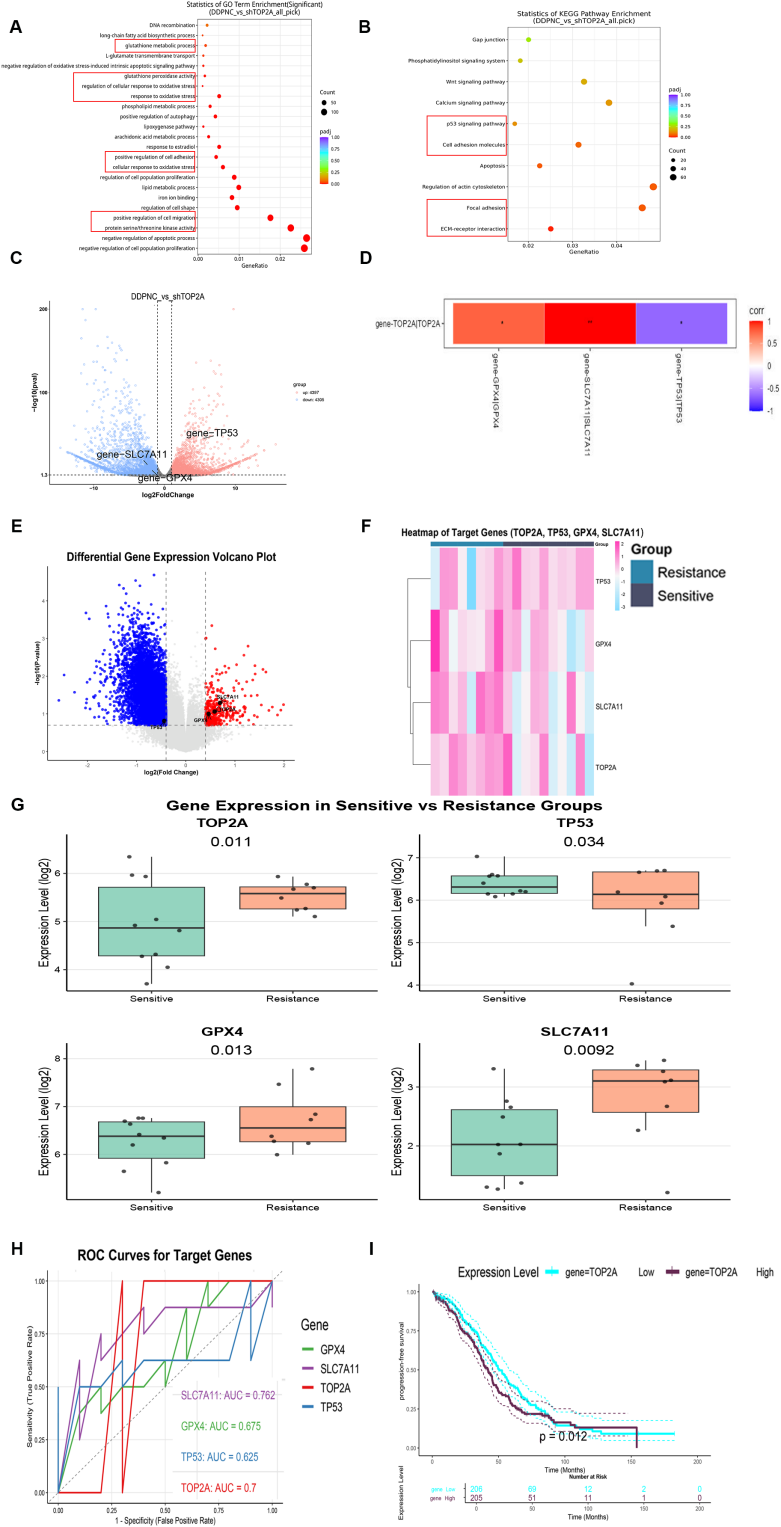
Mechanism and expression analysis of top2A in ovarian cancer cisplatin resistance. **(A, B)** GO **(A)** and KEGG **(B)** Enrichment Analysis of DEGs in SKOV3-DDP with TOP2A Knockdown. **(C)** Volcano Plot of DEGs. **(D)** Correlation between TOP2A and the expression of TP53, GPX4, and SLC7A11 genes in SKOV3-DDP cells. **(E-G)** Volcano plot **(E)**, heatmap **(F)**, and boxplot **(G)** of target genes (TOP2A, TP53, GPX4, SLC7A11) expression in cisplatin-resistant and cisplatin-sensitive populations. **(H)** ROC curves for cisplatin resistance diagnosis using TOP2A, TP53, GPX4, and SLC7A11. **(I)** Kaplan-Meier Progression-Free Survival Curves of Ovarian Cancer Patients Stratified by TOP2A Expression. All experiments were performed with 3 independent biological replicates (n=3), and each biological replicate included 3 technical repeats. Data are shown as mean ± SD. Statistical significance: *p < 0.05, **p < 0.01, ***p < 0.001.

To clarify the mechanism by which TOP2A induces cisplatin resistance in OC, RNA-Seq was performed on TOP2A-silenced SKOV3-DDP cells. Gene Ontology (GO) enrichment analysis indicated significant enrichment in terms related to lipid metabolism regulation, oxidative stress response, glutathione metabolic processes, iron ion binding, positive regulation of oxidative stress-induced cell death, and regulation of protein serine/threonine kinase activity (**Figure 4A**). Furthermore, TOP2A knockdown significantly downregulated the expression of ferroptosis-related proteins GPX4 and SLC7A11 (**Figures 4C, D**), suggesting a close association with the ferroptosis pathway and indicating that TOP2A may regulate cisplatin resistance in OC by suppressing ferroptosis. To further verify the correlation between gene expression and cisplatin resistance phenotype, we performed differential gene expression analysis on cisplatin-sensitive and cisplatin-resistant sample groups via bioinformatics approaches. The volcano plot of differential gene expression (**Figure 4E**) showed a large number of significantly differentially expressed genes between the two groups. Red dots represented significantly upregulated genes, blue dots represented significantly downregulated genes and gray dots represented genes with no significant difference. This indicated substantial differences in gene expression profiles between the sensitive and resistant groups. Specifically, TOP2A, GPX4 and SLC7A11 were upregulated while TP53 was downregulated in the resistant group. The heatmap of target gene expression (**Figure 4F**) clearly showed the expression clustering characteristics of TOP2A, TP53, GPX4 and SLC7A11 across different samples. Based on gene expression patterns, the samples could be clearly divided into subgroups consistent with the sensitive/resistant grouping. These target genes exhibited specific expression differences between the two groups. This suggests a close correlation between their expression and the cisplatin resistance phenotype. Results of intergroup difference analysis using gene expression boxplots (**Figure 4G**) further confirmed key findings. The expression levels of TOP2A (P = 0.011), GPX4 (P = 0.013) and SLC7A11 (P = 0.0092) were significantly higher in the resistant group than in the sensitive group. TP53 expression (P = 0.034) was significantly lower in the resistant group. These results consistently validated the previous conclusions. From the perspective of clinical application value, receiver operating characteristic (ROC) curve analysis (**Figure 4H**) showed differences in the diagnostic efficacy of each target gene for cisplatin resistance. SLC7A11 exhibited the strongest diagnostic ability (AUC = 0.762). TOP2A showed moderate diagnostic efficacy (AUC = 0.700). GPX4 (AUC = 0.675) and TP53 (AUC = 0.625) also had discriminative ability (AUC>0.5) but with lower efficacy than the former two.For survival prognosis analysis, Kaplan-Meier survival curves (**Figure 4I**) showed a key result. The progression-free survival (PFS) curve of ovarian cancer (OV) patients in the high TOP2A expression group was significantly lower than that in the low TOP2A expression group. The Log-rank test confirmed that the difference in PFS between the two groups was statistically significant (P = 0.012). This suggests a close correlation between high TOP2A expression and shorter PFS in ovarian cancer patients.

### TOP2A silencing triggers ferroptosis in cisplatin-resistant OC cells

**Figure 5:**
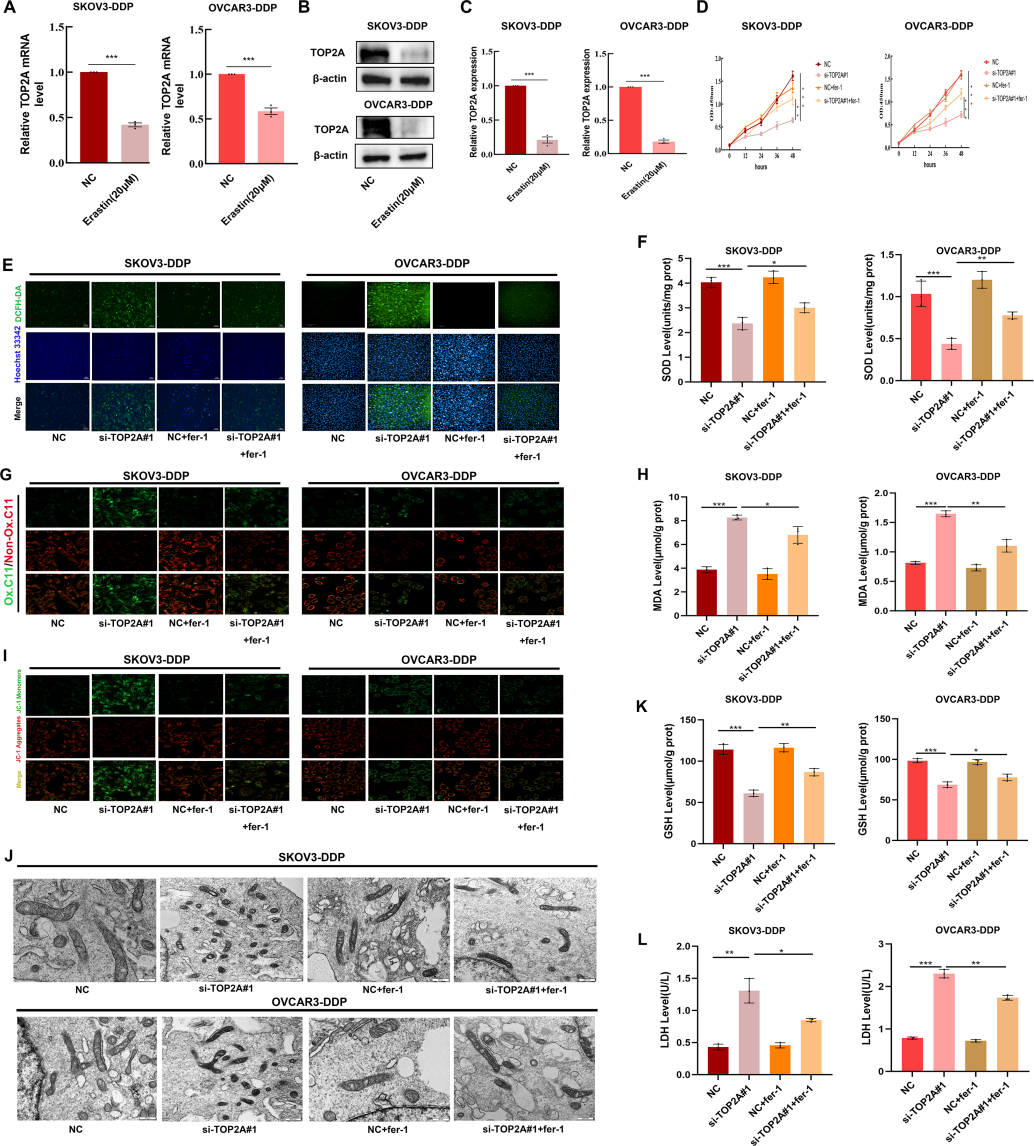
Silencing TOP2A promotes ferroptosis in Cisplatin-Resistant OC Cell. **(A, B)** TOP2A expression levels in SKOV3-DDP and OVCAR3-DDP cells after 24h treatment with 20μM Erastin were detected by qPCR **(A)** and WB **(B)**. **(C)** Quantitative analysis of TOP2A expression levels from **(B)**. **(D)** The effects of TOP2A knockout or combined treatment with 1μM fer-1 on the proliferation of SKOV3-DDP and OVCAR3-DDP cells.**(E-L)**In SKOV3-DDP and OVCAR3-DDP cells, the effects of TOP2A knockdown or combined treatment with 1μM fer-1 on the following parameters were observed: **(E)** ROS levels; **(F)** SOD levels; **(G)** LPO levels; **(H)** MDA levels; **(I)** MMP levels; **(J)** mitochondrial morphology; **(K)** GSH levels; **(L)** LDH levels.All experiments were performed with 3 independent biological replicates (n=3), and each biological replicate included 3 technical repeats. Data are shown as mean ± SD. Statistical significance: *p < 0.05, **p < 0.01, ***p < 0.001.

To explore the relationship between TOP2A and ferroptosis, ferroptosis was induced using the ferroptosis inducer erastin. TOP2A expression levels were detected through qPCR and WB assays.The results showed a significant decrease in TOP2A expression during ferroptosis induction (**Figures 5A-C**). This further confirms the intimate link between TOP2A and ferroptosis. Subsequently, SKOV3-DDP and OVCAR3-DDP cells were treated with the ferroptosis inhibitor Fer-1. The results revealed that Fer-1 effectively reversed the proliferation inhibition induced by TOP2A knockdown (**Figure 5D**), indicating that TOP2A knockdown promotes ferroptosis in cisplatin-resistant OC cells.Since reactive oxygen species (ROS) and superoxide dismutase (SOD) are key markers of oxidative stress (33), intracellular ROS and SOD levels were further measured in SKOV3-DDP and OVCAR3-DDP cells. TOP2A knockdown significantly elevated intracellular ROS levels and reduced SOD levels, and these effects were effectively reversed by fer-1 (**Figures 5E, F**). Lipid peroxidation (LPO) is a core driver of ferroptosis. Its end product malondialdehyde (MDA) was additionally evaluated (34). TOP2A knockdown significantly increased LPO and intracellular MDA levels, and these changes were significantly alleviated by fer-1 (**Figures 5G, H**). Mitochondrial membrane potential (MMP) is a key indicator for evaluating mitochondrial damage during ferroptosis. MMP was detected using the fluorescent probe JC-1 (35), and results showed that TOP2A knockdown reduced MMP levels, an effect effectively reversed by fer-1 (**Figure 5I**). Morphologically, mitochondria undergoing ferroptosis typically exhibit significant shrinkage and cristae loss (36). Transmission electron microscopy showed that mitochondria in TOP2A-knockdown SKOV3-DDP and OVCAR3-DDP cells were significantly smaller with reduced cristae, and this damage was effectively alleviated by fer-1 (**Figure 5J**). Furthermore, Furthermore, glutathione (GSH) and lactate dehydrogenase (LDH) were measured (37, 38). GSH reflects early events of ferroptosis. LDH reflects late events of ferroptosis.TOP2A knockdown reduced GSH levels and increased LDH release. These effects were significantly reversed by fer-1 (**Figures 5K, L**). Taken together, these findings demonstrate that silencing TOP2A enhances ferroptosis in cisplatin-resistant OC cells.

### TOP2A silencing promotes ferroptosis via TP53/GPX4/SLC7A11 and inhibits EMT in cisplatin-resistant OC cells

**Figure 6:**
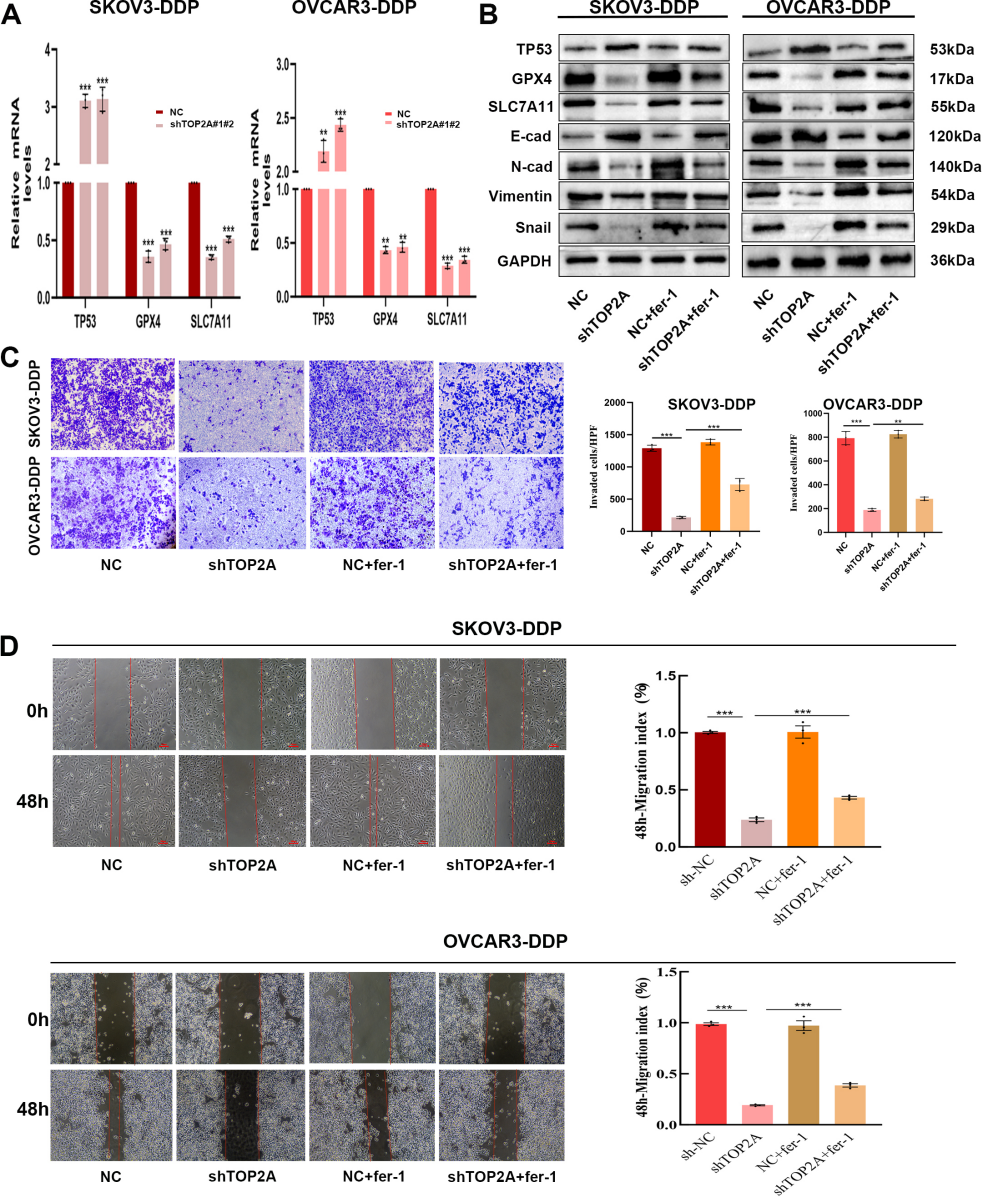
TOP2A knockdown Inhibits EMT in Cisplatin-Resistant OC via P53/GPX4/SLC7A11-Mediated Ferroptosis.**(A)** TP53, GPX4, and SLC7A11 expression levels in TOP2A-knockdown SKOV3-DDP and OVCAR3-DDP cells were detected by qPCR. **(B)** Effects of TOP2A knockdown combined with fer-1 treatment on the expression of TP53, GPX4, SLC7A11 and EMT-related proteins (E-Cadherin, N-Cadherin, Vimentin, Snail) in SKOV3-DDP and OVCAR3-DDP cells. **(C, D)** Effects of TOP2A knockdown combined with Fer-1 treatment on SKOV3-DDP and OVCAR3-DDP cells: **(C)** Invasive capacity detected by Transwell assay; **(D)** Migratory capacity detected by wound healing assay.All experiments were performed with 3 independent biological replicates (n=3), and each biological replicate included 3 technical repeats. Data are shown as mean ± SD. Statistical significance: **p < 0.01, ***p < 0.001.

To clarify the mechanisms by which TOP2A regulates cisplatin resistance in OC, GO enrichment analysis of RNA-Seq data identified strong associations with cell adhesion (**Figure 4A**). KEGG enrichment analysis highlighted the P53 signaling pathway, cell adhesion molecules, focal adhesions, and extracellular matrix (ECM) interactions (**Figure 4B**). Furthermore, TOP2A knockdown was found to reduce GPX4 and SLC7A11 expression while increasing TP53 expression (**Figures 4C, D**). We validated the expression of TP53,GPX4 and SLC7A11, genes in shTOP2A-knockdown SKOV3-DDP and OVCAR3-DDP cells via qPCR, and the results were consistent with RNA-seq data (**Figure 6A**). Subsequently, we detected the expression levels of TP53, GPX4, SLC7A11 proteins, and epithelial-mesenchymal transition (EMT)-related proteins (E-Cadherin, N-Cadherin, Vimentin, Snail) in cisplatin-resistant cells after shTOP2A knockdown using WB.The results showed that after TOP2A knockdown, the expression of TP53 and E-Cadherin was significantly upregulated, while the expression of GPX4, SLC7A11, N-Cadherin, Vimentin, and Snail was significantly downregulated.After treatment with the ferroptosis inhibitor fer-1, the aforementioned changes in protein expression were significantly reversed, but not fully restored to the original levels(**Figure 6B**). Transwell invasion assays showed that the invasive capacity of TOP2A-silenced cisplatin-resistant OC cells was significantly restored following treatment with the fer-1 (**Figure 6C**). Furthermore, scratch wound healing assays further confirmed that fer-1 could restore the migratory ability of these resistant cells (**Figure 6D**). These findings indicate that silencing TOP2A may inhibit the EMT process in OC cells by inducing ferroptosis.

### TP53 is a key target of TOP2A and can regulate the ferroptosis process in cisplatin-resistant OC cells

**Figure 7:**
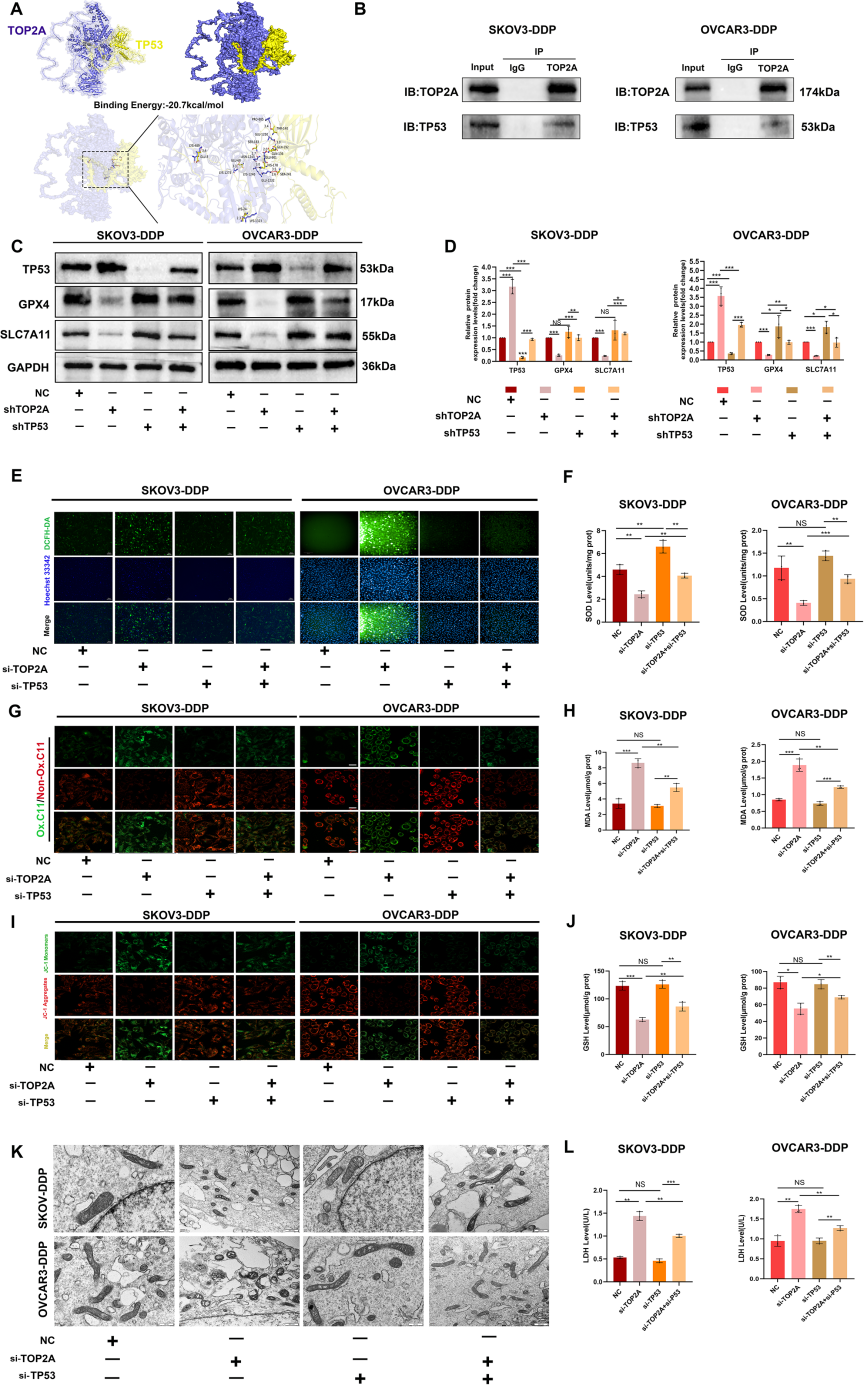
TP53 silencing can reverse ferroptosis induced by TOP2A silencing. **(A )**Molecular docking analysis of TOP2A and TP53 Proteins. **(B)** Co-immunoprecipitation of TOP2A and TP53 in SKOV3-DDP and OVCAR3-DDP cells. **(C)** Silencing TP53 rescued the effects of TOP2A silencing on the expression of TP53, GPX4, and SLC7A11 in SKOV3-DDP and OVCAR3-DDP cells. **(D)** Quantitative analysis of the proteins expression levels from **(C)**. **(E-L)** Silencing TP53 rescued the effects of TOP2A silencing on the following parameters in SKOV3-DDP and OVCAR3-DDP cells: ROS levels **(E)**; SOD levels **(F)**; LPO levels **(G)**; MDA levels **(H)**; MMP levels **(I)**; mitochondrial morphology **(J)**; GSH levels **(K)**; LDH levels **(L)**. All experiments were performed with 3 independent biological replicates (n=3), and each biological replicate included 3 technical repeats. Data are shown as mean ± SD. Statistical significance: *p < 0.05, **p < 0.01, ***p < 0.001. Not Significant:NS.

To explore the interaction potential between TOP2A and TP53, molecular docking was first performed for prediction. The results showed that the binding free energy between TOP2A and TP53 was -20.7 kcal/mol, which was much lower than the well-recognized effective binding threshold of -5.0 kcal/mol (39). This indicated that the two proteins had extremely strong binding ability and excellent system stability. They reached an ideal binding state (**Figure 7A**). Although the surface area of their interaction region was small, this phenomenon was associated with the relatively small overall structure of the proteins. The surfaces of the two proteins formed contacts through multiple sites. Meanwhile, there were multiple hydrogen bond interactions between key amino acid residues. These factors significantly enhanced the stability of the complex. They also collectively supported the high-intensity binding between TOP2A and TP53.Co-IP assay further validated the endogenous interaction between TOP2A and TP53 (**Figure 7B**). To further investigate the role of TP53 in cisplatin-resistant OC cells, we knocked out TP53 in SKOV3-DDP and OVCAR3-DDP cells and verified the knockout efficiency. Results showed that although the expression of ferroptosis-related proteins GPX4 and SLC7A11 in SKOV3-DDP and OVCAR3-DDP cells did not change significantly after TP53 knockdown, their expression levels significantly restored the abnormal expression patterns of GPX4 and SLC7A11 induced by TOP2A knockout (**Figures 7C, D**). As shown in **Figures 7E-L**, we further observed that compared with the TOP2A-silenced group, TP53 silencing significantly attenuated ferroptosis-induced increases in LPO and MDA levels, elevated MMP levels, and restored the morphological features (size and number) of mitochondrial cristae. Additionally, GSH levels were increased, while LDH levels were decreased. These results indicate that TP53 silencing exerts no significant protective effect on cells in the absence of ferroptosis.however, it can markedly reverse TOP2A knockdown-induced ferroptosis in cisplatin-resistant cells.

### TP53 is a key target of TOP2A and regulates EMT in cisplatin-resistant OC cells

**Figure 8:**
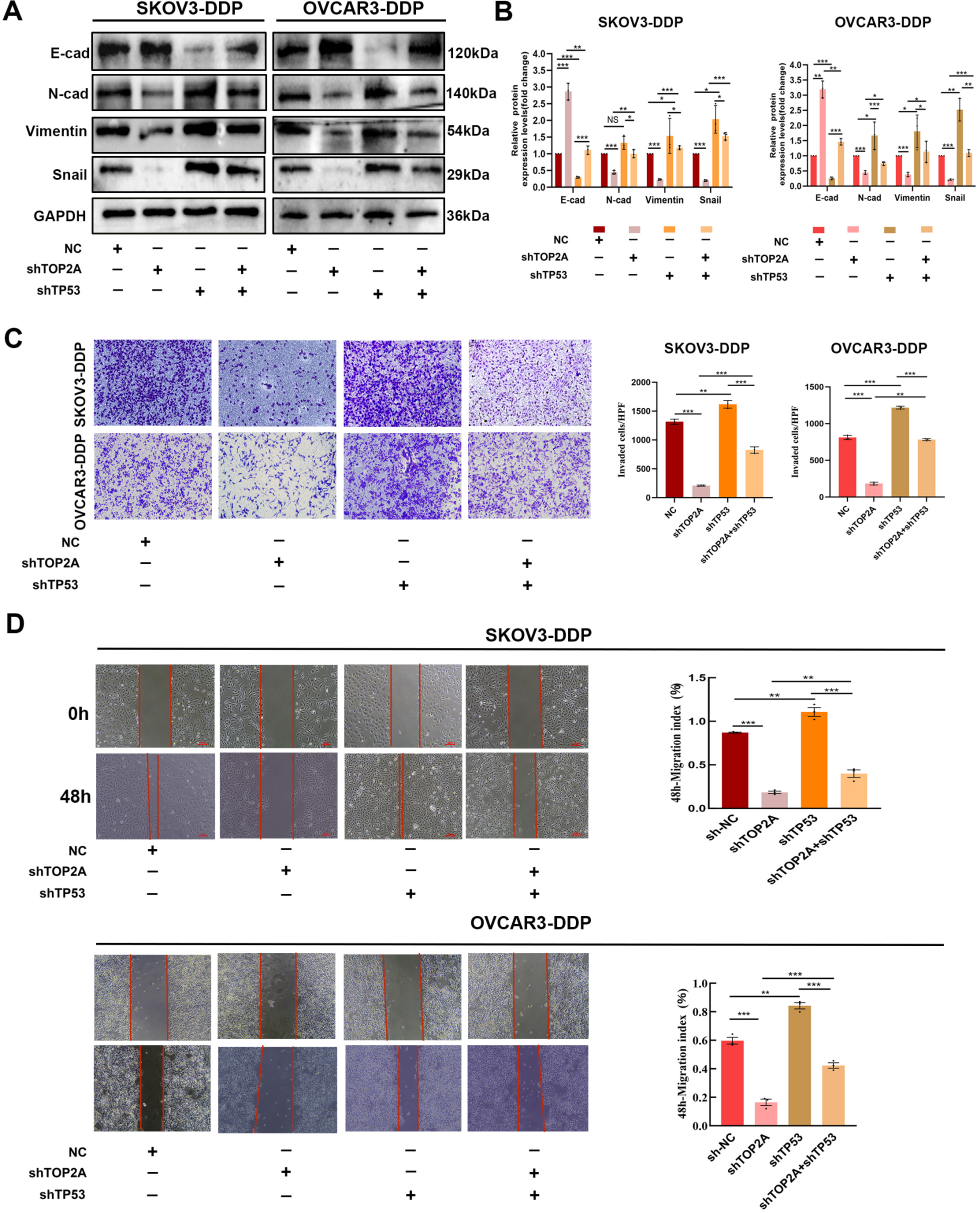
TP53 silencing can reverse EMT induced by TOP2A silencing. **(A)** Silencing TP53 rescued the effects of TOP2A silencing on the expression of E-Cadherin, N-Cadherin, Vimentin, Snail in SKOV3-DDP and OVCAR3-DDP cells. **(B)** Quantitative analysis of the proteins expression levels from **(A)**. **(C, D)** Silencing TP53 rescued the effects of TOP2A silencing on the following parameters in SKOV3-DDP and OVCAR3-DDP cells: Invasive capacity detected by Transwell assay **(C)**; Migratory capacity detected by wound healing assay **(D)**. All experiments were performed with 3 independent biological replicates (n=3), and each biological replicate included 3 technical repeats. Data are shown as mean ± SD. Statistical significance: *p < 0.05, **p < 0.01, ***p < 0.001. Not Significant:NS.

Subsequently, we investigated the association between TP53 and EMT in SKOV3-DDP and OVCAR3-DDP cells. As shown in **Figures 8A, B**, compared with the NC group, TP53 silencing significantly inhibited the expression of E-Cadherin and promoted the expression of N-Cadherin, Vimentin, and Snail in cisplatin-resistant SKOV3-DDP and OVCAR3-DDP cells, suggesting that TP53 may be closely associated with the EMT process. Furthermore, compared with the TOP2A-silenced group, TP53 silencing reversed the changes in the expression of EMT-related proteins induced by TOP2A silencing.Transwell invasion assays and scratch wound healing assays further confirmed that TP53 knockdown not only significantly enhanced the invasive and migratory capacities of cisplatin-resistant ovarian cancer cells but also reversed the inhibition of the EMT process induced by TOP2A knockdown (**Figures 8C, D**). These results indicate that TP53 plays a crucial role in the TOP2A-mediated EMT process in cisplatin-resistant OC cells.

### The promoting effect of TOP2A on cisplatin resistance in ovarian cancer *in vivo*

**Figure 9:**
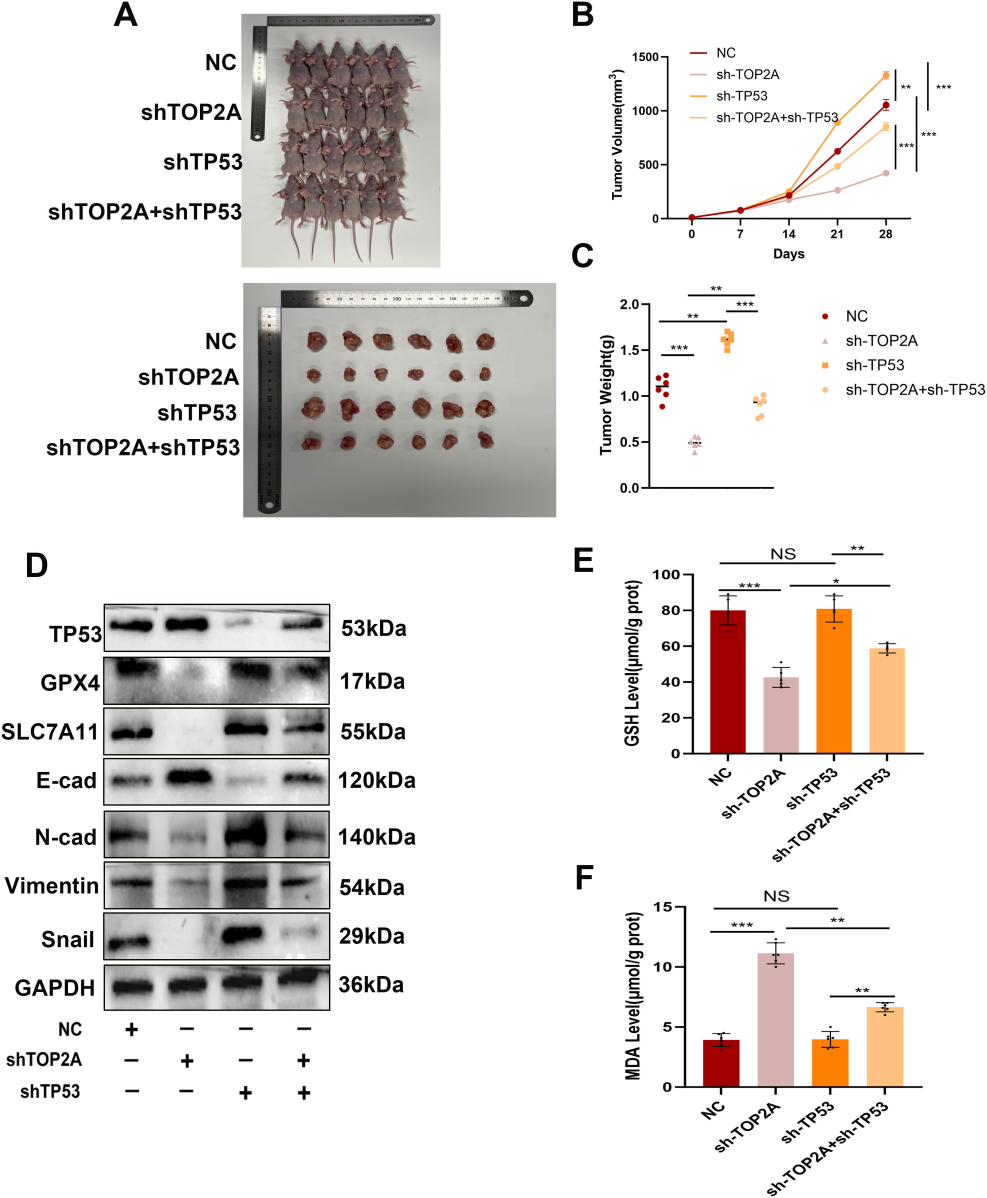
Inhibiting TOP2A can promote ferroptosis and suppress the EMT process in Cisplatin-Resistant OC *in vivo* via TP53. **(A-C)** Measurement of weight **(A, B)** and volume **(C)** of xenografted animals. **(D)** WB analysis showed the expression levels of TP53, GPX4, SLC7A11, E-cadherin, N-cadherin, Vimentin, and Snail in xenografted tumors with TOP2A or TP53 knockdown. **(E, F)** Detection of GSH levels **(E)** and MDA levels **(F)** in xenografted tumors.All experiments were performed with 3–6 independent biological replicates (n=3-6), and each biological replicate included 3 technical repeats. Data are shown as mean ± SD. Statistical significance: *p < 0.05, **p < 0.01, ***p < 0.001. Not Significant:NS.

Based on *in vitro* findings, we established a nude mouse xenograft model of OC-resistant cells with TOP2A and TP53 knockout (**Figures 9A-C**). During cisplatin treatment, TOP2A knockdown significantly inhibited tumor growth and reduced tumor weight, while TP53 knockdown promoted tumor growth and reversed the effect of TOP2A knockdown. These results indicate that TOP2A knockdown can enhance the sensitivity of OC to cisplatin, whereas TP53 knockdown reduces such sensitivity.Furthermore, through WB analysis of tumor tissues, we confirmed conclusions consistent with *in vitro* experiments: TOP2A silencing can induce ferroptosis by regulating the expression of TP53/GPX4/SLC7A11, thereby inhibiting the EMT process (**Figure 9D**). Further detection of ferroptosis-related indicators in tissues revealed that TOP2A knockdown reduced GSH levels and increased MDA levels *in vivo*; in contrast, TP53 knockdown partially restored the decrease in GSH levels induced by TOP2A knockdown and alleviated the increase in MDA levels (**Figures 9E, F**). In summary, these results demonstrate that TOP2A regulates ferroptosis and the EMT process in cisplatin-resistant OC through its interaction with TP53 *in vivo*.

## Discussion

OC ranks among the most prevalent malignancies in the female reproductive system. HGSOC represents its most aggressive subtype (40). A critical clinical challenge in HGSOC management is the development of cisplatin resistance, which frequently leads to disease relapse, treatment failure, and mortality (41). These findings underscore an urgent need for novel therapeutic strategies and molecular targets. Such strategies and targets aim to improve outcomes in patients with platinum resistance. Accumulating evidence has highlighted the pivotal role of TOP2A in cancer progression (42–45). As a validated target for chemotherapeutics such as doxorubicin, etoposide, and methotrexate (46, 47), the expression patterns of TOP2A in OC, particularly the differential expression between primary and recurrent tumors, have been associated with chemotherapeutic response and resistance (48, 49). Notably, integrating TOP2A inhibitors into platinum-based regimens has shown promise in enhancing chemosensitivity and mitigating resistance in recurrent OC, while TOP2A quantification in both tumor and stromal compartments serves as a prognostic biomarker for treatment outcomes (49). However, the molecular pathways through which TOP2A mediates platinum resistance in OC remain incompletely defined. In this study, we demonstrated that TOP2A is upregulated in HGSOC tissues, with its expression levels closely related to tumor grade, stage, and lymph node metastasis, consistent with previous reports (6, 48, 49).Additionally, TOP2A expression is also significantly upregulated in platinum-resistant OC cell lines. We conducted transcriptome analysis of cisplatin-resistant cells. We also performed bioinformatics analysis on cisplatin-resistant and cisplatin-sensitive OC populations using the GEO database. Combined with *in vitro* and *in vivo* functional validation, these analyses further confirmed that high TOP2A expression is associated with poorer PFS in patients. They also confirmed that high TOP2A expression serves as an independent prognostic factor for the diagnosis of cisplatin-resistant OC.Meanwhile, we identified TOP2A as a therapeutic target for platinum-resistant OC. We also uncovered a novel mechanism for the first time. Knocking down TOP2A promotes ferroptosis and EMT in cells. This process occurs through the TP53 signaling pathway. It further reverses cisplatin resistance in OC.

Initial experiments revealed that TOP2A knockdown significantly impaired the proliferative capacity of cisplatin-resistant OC cells. When combined with cisplatin treatment, sh-TOP2A-transfected cisplatin-resistant cells exhibited marked reductions in proliferation, invasion, and migration, indicating a significant restoration of cisplatin sensitivity. These findings highlight the critical role of TOP2A in mediating cisplatin resistance in OC.

RNA-seq analysis revealed differential gene expression patterns associated with shRNA-mediated TOP2A knockdown in cisplatin-resistant OC cells. Functional enrichment analysis showed that these genes were significantly enriched in pathways related to lipid metabolism regulation, oxidative stress response, glutathione metabolism, iron ion binding, regulation of oxidative stress-induced cell death, and protein kinase activity regulation. These enriched pathways are closely related to ferroptosis.Ferroptosis is a novel form of cell death, distinct from apoptosis, characterized by the harmful accumulation of lipid peroxides on the cell membrane, typically activated by oxidative stress caused by glutathione depletion, hemin accumulation, and/or reactive lipid species. Ferroptosis holds great potential in cancer therapy by inhibiting tumor formation and enhancing tumor immune responses (50, 51).The inhibitory network of ferroptosis is coordinately regulated by multiple pathways, among which the GSH-GPX4 axis, as the core pathway, maintains cell membrane homeostasis by scavenging lipid ROS (52).Notably, inhibiting ferroptosis can enhance chemoresistance in cancer cells, while activating ferroptosis can restore their chemosensitivity (53, 54).Previous studies have identified several key pathways regulating ferroptosis in OC, such as the USP43-FASN-HIF1α-SLC7A11 axis (a negative regulator of ferroptosis) and the NRF2-FSP1 pathway (a protective barrier against ferroptosis (55, 56).Beyond these, VIPAS39 has also been reported to facilitate tumor cells in evading immunogenic ferroptosis and sustaining proliferative advantages by delivering ACSL4 protein via exosomes, which in turn reduces the abundance of lipid peroxidation substrates (57).Collectively, these studies have established that systematic intervention in the core regulatory network of ferroptosis is an effective strategy to overcome chemotherapy resistance in OC (58).Mitochondria are key regulatory hubs for ferroptosis, and their metabolic status directly influences the process of lipid peroxidation (59).Studies have shown that inhibiting the mitochondrial tricarboxylic acid cycle or electron transport chain can reduce hyperpolarization of mitochondrial membrane potential, lipid peroxidation, and ferroptosis (60).Additionally, under hypoxic conditions, mitochondrial fission increases ROS levels in ovarian cancer cells, thereby contributing to cisplatin resistance (61).This “mitochondria-oxidative stress-resistance” regulatory axis provides a metabolic basis for the association between TOP2A and ferroptosis. Based on the aforementioned mechanisms, we further validated the regulatory relationship between TOP2A and ferroptosis in cisplatin-resistant OC using multidimensional experiments. First, functional validation experiments demonstrated that the ferroptosis inducer Erastin downregulated TOP2A expression. This finding aligns with the mechanism identified in hepatocellular carcinoma research, where phillyrin promotes ferroptosis by inhibiting the TOP2A-JAK2/STAT3 axis (29), suggesting that TOP2A may act as a conserved molecular node linking ferroptosis regulation and chemoresistance. To explore the downstream mechanisms, RNA sequencing analysis showed that the expression of the key ferroptosis regulators GPX4 and SLC7A11 significantly decreased following TOP2A knockdown. GPX4 maintains cell membrane stability by catalyzing the clearance of lipid reactive oxygen species via GSH; SLC7A11 is responsible for transporting extracellular cystine to supply the raw materials for GSH synthesis (62–64). The loss of GPX4 and SLC7A11 expression leads to an imbalance in lipid peroxidation, ultimately resulting in the characteristic membrane damage associated with ferroptosis. This result confirms that TOP2A can influence the ferroptosis process by regulating the GPX4/SLC7A11 pathway. Subsequently, we performed functional rescue experiments using the ferroptosis inhibitor fer-1 (65). The results showed that TOP2A knockdown significantly increased intracellular ROS levels, induced mitochondrial contraction, increased membrane density, and reduced or disappeared cristae, along with decreased GSH and SOD levels, reduced mitochondrial membrane potential, and significantly elevated MDA and LPO levels. Fer-1 treatment partially reversed these morphological changes, including restoring GSH levels, improving mitochondrial morphology, and enhancing antioxidant capacity. These findings indicate that TOP2A promotes cisplatin resistance in OC cells by inhibiting ferroptosis.

Notably, TOP2A knockdown significantly enriched pathways related to cell adhesion molecules and ECM-receptor interactions, which directly regulate cell migration. EMT, a core process in ovarian cancer invasion and metastasis, is activated by ECM remodeling and adhesion molecule rearrangement, contributing to cisplatin resistance (66–68).In this study, TOP2A knockdown significantly reduced the invasive capacity of cisplatin-resistant cells, accompanied by characteristic changes in EMT markers: increased E-cad protein expression and decreased expression of N-cad, vimentin, and Snail. These findings indicate that TOP2A knockdown inhibits the EMT process in resistant cells. Mechanistically, ferroptosis and EMT are closely interconnected through a cross-regulatory network, and inhibiting ferroptosis can promote the EMT process (69). Fer-1 can reverse drug-induced EMT in esophageal cancer cells (70). Consistent with the aforementioned findings, our study demonstrated that the ferroptosis inhibitor fer-1 significantly reversed the inhibitory effects of TOP2A knockdown on EMT, confirming that promoting ferroptosis is a necessary intermediate step for TOP2A to inhibit EMT. This regulatory pattern is supported by the enrichment of the TP53 pathway in transcriptome analysis. Although TP53 is not a canonical regulator of EMT, it can act as a shared regulator of ferroptosis and EMT. TP53 activation can block EMT by inhibiting Snail transcription (71),and TOP2A knockdown enhances TP53 activity via the TP53 signaling axis.

TP53 is the most frequently mutated tumor suppressor gene in cancers, and its wild-type form can inhibit tumor growth through multiple mechanisms (72).In the context of cisplatin resistance, TP53 mutations exert dual regulatory effects: on one hand, TP53 mutations can promote resistance phenotypes in mediastinal germ cell tumors (73)and high-grade serous ovarian cancer (74), patients harboring functional TP53 mutations exhibit the lowest cisplatin sensitivity and the poorest platinum-free survival prognosis (75). On the other hand, nanoparticles targeting mutant TP53 can degrade mutant p53 and induce endoplasmic reticulum stress, thereby restoring cisplatin sensitivity in non-small cell lung cancer (NSCLC) (76). These findings indicate that TP53 status is closely associated with platinum-based chemotherapy sensitivity.In the regulation of ferroptosis, TP53 transcriptionally represses the expression of SLC7A11 (a subunit of the cystine/glutamate antiporter), reducing extracellular cystine uptake and disrupting the supply of raw materials for GSH synthesis, ultimately inducing lipid peroxidation and increasing cellular sensitivity to ferroptosis (36, 77, 78). Notably, our bioinformatics analysis of cisplatin-resistant and cisplatin-sensitive OC samples from the GEO database confirmed previous findings. Specifically, in the cisplatin-resistant group, the expression levels of TOP2A, GPX4, and SLC7A11 were significantly increased, while the expression of TP53 was significantly decreased. Moreover, TOP2A could serve as an effective diagnostic and prognostic gene, and its high expression was associated with poorer PFS. Subsequently, this mechanism was verified through molecular docking combined with *in vitro* experiments. TOP2A knockdown downregulates SLC7A11 and GPX4 expression via the TP53 signaling axis, triggering ferroptosis-associated characteristic membrane damage. In EMT regulation, p53 can target EMT master regulators through miRNA-dependent mechanisms. For instance, p53 activation upregulates miR-34a, which inhibits EMT by targeting Zeb1 (71).MCOLN1/TRPML1-induced autophagy inhibition suppresses cancer metastasis by activating the ROS-mediated TP53/p53 pathway (79).These findings provide a theoretical basis for TP53 acting as a hub in the crosstalk between ferroptosis and EMT. In cisplatin-resistant cells, simultaneous knockdown of TOP2A and TP53 revealed that TP53 deficiency not only reversed TOP2A knockdown-induced changes in GPX4 and SLC7A11 expression and ferroptosis-related phenotypes but also reversed the cells’ invasive/migratory capabilities and EMT-related protein expression. This confirms that TOP2A mediates ferroptosis via the TP53/GPX4/SLC7A11 axis, thereby inhibiting EMT and reversing cisplatin resistance. Finally, we established a nude mouse subcutaneous xenograft model, which further confirmed that TOP2A knockdown enhances the sensitivity of resistant cells to cisplatin, likely through the TP53/GPX4/SLC7A11 axis. These results provide more comprehensive evidence for targeting TOP2A in the treatment of cisplatin-resistant OC.

This study still has two limitations that require further improvement. First, although the analysis was conducted using a drug-resistant population database, validation with an independent cohort of clinical drug-resistant samples is lacking. The existing data cannot fully support details such as the expression heterogeneity of TOP2A in drug-resistant patients and the dynamic changes in pathway activity. Additional clinical samples will be needed for validation in subsequent studies.Second, mechanism validation was performed only using TP53 mutant cell lines. TP53 wild-type ovarian cancer cells were not included in the analysis. This makes it impossible to clarify whether the role of TOP2A is associated with the TP53 mutation background. Validation with wild-type models will be required in future studies.

In conclusion, this study clarified the regulatory role of TOP2A in cisplatin resistance of ovarian cancer. It also elucidated the specific mechanism by which TOP2A affects cellular ferroptosis through the TP53 signaling pathway, thereby inhibiting EMT. Specifically, TOP2A is significantly overexpressed in cisplatin-resistant tissue cells. Its high expression level is closely associated with poor prognosis in patients and can serve as an effective diagnostic gene. At the mechanistic level, knocking down TOP2A can upregulate the expression of the tumor suppressor gene TP53. It can simultaneously downregulate the expression of GPX4 and SLC7A11. This further induces ferroptosis in cisplatin-resistant cells. Ultimately, it inhibits the EMT process and the malignant phenotype of tumor cells.Based on these findings, intervention strategies targeting TOP2A or combining with TP53 agonists provide a potential direction for the treatment of cisplatin-resistant ovarian cancer and the improvement of patient prognosis.

## Data Availability

The original contributions presented in the study are publicly available. This data can be found here: http://www.ncbi.nlm.nih.gov/bioproject/1314868 (BioProject ID: PRJNA1314868).
