# Supplementary material for: Knockdown of TOP2A reverses cisplatin resistance in ovarian cancer by inhibiting EMT via ferroptosis mediated by the TP53/GPX4/SLC7A11 axis
Source: Front Immunol. 2025 Oct 29;16:1675373. doi: 10.3389/fimmu.2025.1675373 (PMC12604993; doi:10.3389/fimmu.2025.1675373)
Supplement: Supplementary file 1 [file Table1.docx]

**Table S1.The target sequence**

| **targets** | **Sequence(5’to3’)** |
| --- | --- |
| siTOP2A#1(siTOP2A) | CUAGUCCACGAUACAUCUUUA |
| siTOP2A#2 | CUUCUAGUAACUAUGAUGAUG |
| shTOP2A#1(shTOP2A) | CTAGTCCACGATACATCTTTA |
| shTOP2A#2 | CTTCTAGTAACTATGATGATG |
| siTP53#1(siTP53) | CGGCGCACAGAGGAAGAGAAU |
| siTP53#2 | AGAUGUUCCGAGAGCUGAAUG |
| shTP53#1(shTP53) | CGGCGCACAGAGGAAGAGAAT |
| shTP53#2 | AGATGTTCCGAGAGCTGAATG |

**Table S2.Sequences of primers used quantitative real-time PCR.**

| **Gene** | **Forward primer (5’ to 3’)** | **Reverse primer (5’ to 3’)** |
| --- | --- | --- |
| TOP2A | FAAGTGTCACCATTGCAGCCT | TGTCTGGGCGGAGCAAAATA |
| TP53 | CCTCTCCCCAGCCAAAGAAG | CTTCAGGTGGCTGGAGTGAG |
| SLC7A11 | CTCCTGCTTTGGCTCCATGA | CAGCTGGTAGAGGAGTGTGC |
| GPX4 | CAGTGAGGCAAGACCGAAGT | CCGAACTGGTTACACGGGAA |
| GAPDH | CAGGAGGCATTGCTGATGAT | GAAGGCTGGGGCTCATTT |

**Table S3 Information on antibodies used in Western Blotting**

| Antibodies | Catalog number | Company |
| --- | --- | --- |
| TOP2A(1:2000) | 66541-1-lg/p11388 | Proteintech/Abmart |
| β-actin(1:80000) | 60008-1-lg | Proteintech |
| GPX4(1:2000) | [T56959](http://www.ab-mart.com.cn/page.aspx?node= 77 &id= 2627" \t "https://www.ab-mart.com.cn/_blank) | Abmart |
| SLC7A11(1:2000) | T57046 | Abmart |
| TP53(1:2000) | TA0879 | Abmart |
| E-Cadherin(1:2000) | TA0131 | Abmart |
| N-Cadherin(1:2000) | T55015 | Abmart |
| Vimentin(1:2000) | T55134 | Abmart |
| Snail(1:2000) | TA6032 | Abmart |
| GAPDH(1:5000) | [M20006](http://www.ab-mart.com.cn/page.aspx?node= 59 &id= 984" \t "https://www.ab-mart.com.cn/_blank) | Abmart |
| Goat Anti-Rabbit Mouse IgG-HRP (1:8000 | M21003 | Abmart |
